# Comparative Evaluation of Automatic Detection and Classification of Daily Living Activities Using Batch Learning and Stream Learning Algorithms

**DOI:** 10.3390/jpm15050208

**Published:** 2025-05-20

**Authors:** Paula Sofía Muñoz, Ana Sofía Orozco, Jaime Pabón, Daniel Gómez, Ricardo Salazar-Cabrera, Jesús D. Cerón, Diego M. López, Bernd Blobel

**Affiliations:** 1 Telematics Engineering Research Group, Telematics Department, Universidad del Cauca, Popayán 190002, Colombia; paulasofia@unicauca.edu.co (P.S.M.); ansorozco@unicauca.edu.co (A.S.O.); japabon216@unicauca.edu.co (J.P.); dgomez216@unicauca.edu.co (D.G.); ricardosalazarc@unicauca.edu.co (R.S.-C.); jesusceron@unicauca.edu.co (J.D.C.); dmlopez@unicauca.ed.co (D.M.L.); 2 Medical Faculty, University of Regensburg, 93053 Regensburg, Germany; 3 eHealth Competence Center Bavaria, Deggendorf Institute of Technology, 94469 Deggendorf, Germany; 4 First Medical Faculty, Charles University Prague, 12800 Prague, Czech Republic

**Keywords:** activities of daily living, ADL, human activity recognition, HAR, batch learning, stream learning, algorithm comparison

## Abstract

**Background/Objectives:** Activities of Daily Living (ADLs) are crucial for assessing an individual’s autonomy, encompassing tasks such as eating, dressing, and moving around, among others. Predicting these activities is part of health monitoring, elderly care, and intelligent systems, improving quality of life, and facilitating early dependency detection, all of which are relevant components of personalized health and social care. However, the automatic classification of ADLs from sensor data remains challenging due to high variability in human behavior, sensor noise, and discrepancies in data acquisition protocols. These challenges limit the accuracy and applicability of existing solutions. This study details the modeling and evaluation of real-time ADL classification models based on batch learning (BL) and stream learning (SL) algorithms. **Methods:** The methodology followed is the Cross-Industry Standard Process for Data Mining (CRISP-DM). The models were trained with a comprehensive dataset integrating 23 ADL-centric datasets using accelerometers and gyroscopes data. The data were preprocessed by applying normalization and sampling rate unification techniques, and finally, relevant sensor locations on the body were selected. **Results:** After cleaning and debugging, a final dataset was generated, containing 238,990 samples, 56 activities, and 52 columns. The study compared models trained with BL and SL algorithms, evaluating their performance under various classification scenarios using accuracy, area under the curve (AUC), and F1-score metrics. Finally, a mobile application was developed to classify ADLs in real time (feeding data from a dataset). **Conclusions:** The outcome of this study can be used in various data science projects related to ADL and Human activity recognition (HAR), and due to the integration of diverse data sources, it is potentially useful to address bias and improve generalizability in Machine Learning models. The principal advantage of online learning algorithms is dynamically adapting to data changes, representing a significant advance in personal autonomy and health care monitoring.

## 1. Introduction

The paper is an extended consideration of aspects published in earlier pHealth Proceedings [1,2,3,4]. The Personalized Health (pHealth) paradigm is supported by adaptive, context-aware technologies that dynamically respond to evolving patient behaviors and environmental conditions [1,2]. Aging populations can benefit from pHealth solutions, which support independent living and improve quality of life and autonomy. Activities of Daily Living (ADLs), such as eating, dressing, and moving around, are crucial for assessing a person’s autonomy. Human Activity Recognition (HAR) systems aim to identify ADL automatically. Therefore, it is essential to acquire certain information to perform the automatic classification of an ADL, e.g., the person’s location when performing certain activities and the position of the sensor on the body. In addition, it is crucial to consider the temporal patterns of the activities. We found several works related to machine learning and deep learning for human activity recognition [3,4,5,6,7,8,9,10,11,12]; however, these works show that although there are advances in HAR research, current systems still face limitations in adapting to dynamic environments or evolving patient behaviors—critical shortcomings for personalized health monitoring [13,14].

Previous research on HAR has identified several datasets to train Machine Learning (ML) algorithms [15]. The models addressed a typical classification problem that attempts to identify the activity performed by an individual at a given time. This problem is usually solved using models based on traditional offline learning algorithms—also known as Batch Learning (BL) algorithms—where the training data are available before the model building. However, these models cannot adapt to continuous data changes. The data used to recognize human activities are constantly changing over time due to changes in human behavior, such as differences in the physical characteristics of each person and the health status of individuals, as well as changes in the physical environments where the person inhabits [16].

Moreover, it has recently been found relevant to store and classify ADL directly on smartphones or wearables. This process is called Smartphone-based HAR (SHAR). One of the reasons for performing SHAR is to provide security for the collected data, as it avoids using an external server, which can be vulnerable to cyber-attacks. However, when activity recognition is performed within smartphones or wearables, offline models are critical because classification models must constantly collect and analyze sensor data. Additionally, smartphones and wearables have memory and processing limitations [16].

Online models built using Stream Learning (SL) algorithms, also known as Online Learning algorithms, present more flexibility for evolving data [17]. Unlike offline learning, where training data are provided simultaneously, online learning processes data incrementally, enabling the model to adapt continuously. As a result, online models are well-suited for systems with limited memory resources. In this way, the model incorporates new information for segmenting activity data, which evolves rapidly, detecting changes and adapting the activity models to the latest information. In addition, they require lower computational costs [18]. In this context, using SHAR applications for online learning and classification in SL becomes relevant [19].

This research proposes implementing models based on SL (online learning) and BL (offline learning) algorithms for ADL classification. The model is trained with a robust dataset built in previous work, in which 23 ADL-centric datasets with accelerometers and gyroscopes data were integrated and analyzed. This paper aims to measure and compare the performance of both approaches using specific evaluation metrics such as accuracy, AUC, and F1-score. By comparing SL and BL in ADL classification, we advance toward context-aware systems that recognize what activities are performed and how they evolve, allowing personalized patient monitoring.

The novelty of this study lies in the exhaustive comparison of models built with SL and BL algorithms, considering the adaptability and effectiveness of each approach. In addition, a deployment prototype (mobile application) is presented, which allows the classification of ADLs in real time, offering a practical and innovative approach to detecting and monitoring these activities.

The rest of the article is organized into four sections: Section 2 (Materials and Methods) describes the creation of the dataset, BL and SL modeling and evaluation process, and mobile application deployment to classify ADLs. Section 3 (Results) presents the findings of each phase and compares both algorithms. Section 4 (Discussion) discusses the results, limitations, and future research. Finally, Section 5 (Conclusions) summarizes the key contributions to ADL classification.

## 2. Materials and Methods

For the development and implementation of this research, the Cross Industry Standard Process for Data Mining (CRISP-DM) methodology was selected for its flexibility and adaptability to different domains [20]. This methodology offers a structured and efficient data mining project implementation process. Thanks to a previous study performed by our research team, a comprehensive dataset was configured. The dataset provides precise information for the training and evaluation of the classification models. This dataset was generated during the first phases of CRISP-DM: business understanding, data understanding, and data preparation [21]. This paper complements the work, reporting the remaining phases: modeling BL and SL algorithms, evaluating their performance, and deploying. After describing the dataset, the modeling and evaluation phases are detailed in the next sub-sections, providing a clear overview of the process to achieve the results.

**Figure 1:**
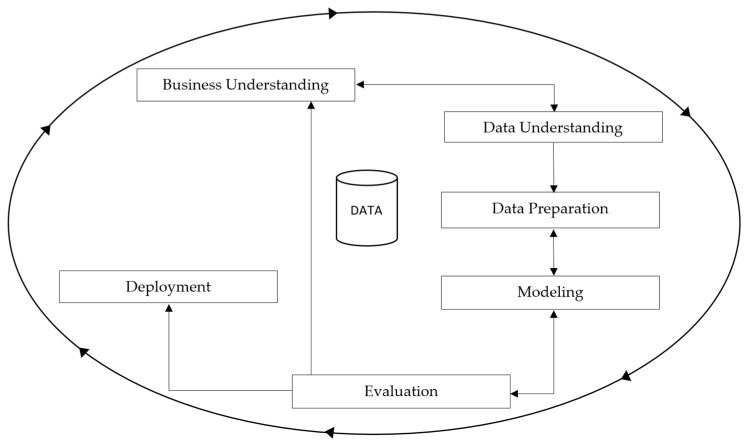
Workflow of the CRISP-DM methodology.

Figure 1 illustrates the CRISP-DM methodology. The flowchart outlines the six key phases of the process—business understanding, data understanding, data preparation, modeling, evaluation, and deployment—demonstrating the approach’s iterative and flexible nature. This visual representation helps clarify the structured workflow followed in this study, from the initial data exploration to the final deployment of the classification models.

### 2.1. Dataset Description

The dataset, named “Mobile System for the Automatic Classification of Activities of Daily Living”, comprises 238,990 samples, 56 different activities, and 52 columns featuring segment number, dataset number, activity number, sensor_type, left_right, location, and 46 columns related to the selected features. Seven features (entropy, average, standard deviation, maximum, minimum, mean, absolute average) were measured for each of the acceleration measurements (Acc) in every axis (x, y, z), as well as for each of the gyroscope measurements (Gyro) in each axis. Thus, there are 21 columns related to Acc and 21 to Gyro. The remaining two selected characteristics (resulting average and magnitude) are general measurements in all directions, resulting in only one value for Acc and another for Gyro for each characteristic, adding another four columns to the 42 mentioned above, totaling 46 columns related to the selected characteristics in the final dataset.

The dataset is available to the public via a link on the Kaggle platform [21].

### 2.2. Modelling

This section details the modeling process performed according to the CRISP-DM methodology, using two approaches, BL and SL, through specific libraries, such as scikit-learn and scikit-multiflow, respectively. The modeling development is organized into three key tasks. Each task includes essential activities to ensure structured and accurate analysis, from identifying suitable models to evaluating results using different datasets and validation strategies.

#### 2.2.1. Selection of Modeling Techniques

The study’s main objective was to compare models trained with BL and SL algorithms using a generated dataset and evaluate their performance under various classification scenarios and metrics. Considering this objective, it is important to determine which algorithms were selected for the evaluation from each type (BL and SL).

This task selected the most suitable models for BL and SL, considering each approach’s specific characteristics and requirements. For example, BL processes the data in complete batches to train the model. Therefore, robustness and the ability to handle large volumes of data were prioritized. While SL uses fractions of the data for training, adjusting to incremental learning, which adapts to the arrival of new data [22], so models for streaming data and adapting to dynamic changes are necessary. The models chosen for each technique are presented in Table 1 and Table 2, where their descriptions and main hyperparameters are included.

#### 2.2.2. Models Pre-Training

Each BL and SL model mentioned in the tables above was trained and tested using the dataset. The pre-training preparation of the BL and SL models differs significantly, as described below.

##### Preparation of BL Models

The process was the same for the seven models in Table 1; the only difference was the classifier used. We started dividing the dataset into independent variables, denoted as X, representing the features, and the dependent variable Y, with the class labels. This splitting is performed using the StratifiedShuffleSplit technique, which ensures a balanced distribution of classes in the training (70%) and test (30%) sets.

Once the splitting was completed, the missing values (“NaN”) were modified by zeros in the training and test sets. In addition, the column ‘Activity’, which identifies the label of the activity performed, was removed as it is irrelevant to the training process. With the dataset prepared, a classifier was initialized (depending on the algorithm used) to train the X-train and Y-train sets.

##### Preparation of SL Models

The preparation of the training phase of the four SL models began by importing the scikit-multiflow library. Also, the preparation of the data included replacing “NaN” values with 0, bearing in mind that SL is an approach trained by mini-batches or fractions of the data; therefore, removing outliers could introduce biases. Subsequently, the dataset was divided into training and test sets, with a ratio of 70 and 30, respectively. This splitting was performed using the stratified splitting technique, which ensures that the proportion of classes in both sets is maintained.

Table 3 presents 4 of the 23 datasets used as data sources in the configuration process of the final integrated dataset. These four datasets were important for evaluating the models developed with the integrated dataset. The following subsections explain how these four datasets were used. It is important to highlight that the datasets with IDs 1 and 2, although initially used as a data source in the integration process, were discarded during the integration process. The datasets with IDs 3 and 4 are part of the integrated dataset.

Two additional sets were generated to assess the reliability of the data: one excluding the dataset with ID 2 (in Table 3) from the integrated dataset and one using only the dataset with ID 2. This decision was made because this dataset occupied a significant weight (78.928%) in the final dataset, which allowed us to assess its impact on the model’s performance.

- Dataset_without_2: This dataset, excluding dataset with ID 2, is used to evaluate the model’s performance. It describes 42 activities, has 50,360 rows, and is 39.7 MB in size.
- Dataset_2: This dataset is used to evaluate the performance of the dataset with ID 2 individually. It describes 30 activities, has 188,630 rows, and is 103 MB in size.

#### 2.2.3. Test Plan Generation

With the three datasets mentioned above, the complete dataset, dataset_without_2, and dataset_2, a test plan was established consisting of preliminary models and refined models.

##### Preliminary Models

Preliminary models incorporate all unified activities to achieve a structured and complete comparison between BL and SL. Two preliminary models were created: those that do not include random values (Preliminary Model WithOut Random Values, PMWORV) and those that do (Preliminary Model With Random Values, PMWRV). Two important aspects were considered in building these preliminary models. Firstly, an initial analysis will be conducted by replicating the BL training and evaluation process in SL to understand the behavior of both approaches and make future decisions based on these findings. Secondly, by checking the behavior of the models, we sought to ensure their impartiality to guarantee that both can make fair and equitable decisions, avoiding biases. Once the above aspects were taken into account, the models were generated as follows:

- PMWORV. These models were ideally trained, which required avoiding any modification to the resulting datasets, thus obtaining as faithful a representation of the original data as possible. The aim is to replicate the BL training and evaluation process in SL. However, the fundamental difference between the two models must be considered. While BL is trained per fragment and evaluated on another fragment, SL is trained and assessed simultaneously on the entire available dataset. Therefore, SL was trained and evaluated using 70% of the dataset to simulate a process similar to that of BL. Subsequently, a “pkl” file was created with this previous training, which was used to retrain and evaluate SL using the remaining 30% of the dataset. In this way, we sought to parallel the nature of BL, which was trained with 70% of the data and evaluated with the remaining 30%.
- PMWRV. These models were designed to be trained and evaluated in an unfavorable environment to ensure fairness. To achieve this, we chose to randomize the values of the three datasets during the training and evaluation process. In the case of SL, the dataset was randomized and then trained and evaluated conventionally, i.e., using the entire dataset. This approach provided more unbiased results, as the model approximated a real environment.
- On the other hand, the dataset for BL was also randomized. However, after multiple tests, it was confirmed that this process did not significantly affect BL due to its batch training. For this reason, cross-validation was proposed for BL.

The preliminary models provided valuable information, revealing that the “dataset_without_2” performed better than the other datasets. It contains less information regarding rows and activities, facilitating classification and improving the model’s performance. The dataset 2 was excluded because it had too much data, so we wanted to prevent overfitting. It was also discarded because the variance in the dataset’s gyroscope data was high. This observation corroborated why the previous cases did not perform as expected and also highlights the importance of carefully selecting datasets for model training.

The evaluation of preliminary models, including both PMWORV and PMWRV, highlights notable differences in computational resource consumption between BL and SL approaches. BL models, particularly Random Forest, demand higher RAM and processing power due to their batch training methodology, making them more suitable for environments with stable data availability but less feasible for real-time applications. Conversely, SL models demonstrate lower resource consumption by processing data incrementally, which optimizes memory usage and computational efficiency. These differences become particularly relevant when analyzing PMWRV models, where randomized datasets introduce additional complexity. SL models maintain adaptability despite data variability, showing efficient memory utilization and faster processing. Meanwhile, BL models remain computationally demanding regardless of dataset structure. These findings underscore the importance of computational cost considerations when selecting a machine learning approach, especially in resource-constrained environments where memory availability and real-time adaptability are crucial.

##### Refined Models

**Figure 2:**
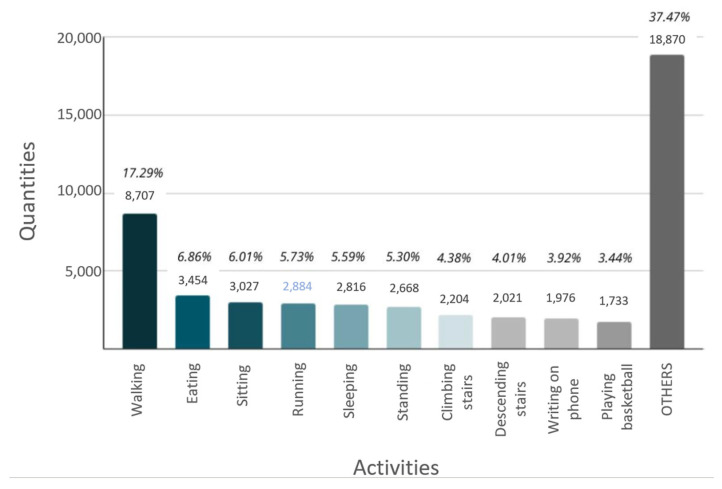
Weight of activities in the dataset_without_2 (integrated dataset without dataset width ID 2).

Contrary to the preliminary models, the refined models did not consider all the collected activities to evaluate the behavior of the BL and SL models in a dataset with fewer activities. The approach of the PMWRV models was considered when selecting the activities in the clean models. Those activities with the highest weight within the dataset that showed the best performance (dataset without 2) were chosen. As a result of this process, the following activities were obtained: walking (6), eating (61), sitting (16), running (9), sleeping (18), standing (17), climbing stairs (13), descending stairs (14), writing on mobile phone (37), playing basketball (59). The remaining activities were included in the “other” category, which was immediately eliminated, as shown in Figure 2. After cleaning the dataset, the “y” axis of the graph in Figure 2 corresponds to the samples of each activity.

Although Figure 2 shows activity 6 (walking) with a weight of 17.29%, significantly higher than other activities, this reflects an initial class imbalance identified during the data exploration phase. This imbalance can negatively affect the classification models by introducing a bias towards the majority classes.

To solve this problem, sampling techniques such as oversampling and under-sampling were applied to balance the class distribution. These techniques adjusted the representation of minority and majority activities by adding or removing samples accordingly. This step ensured that all activities selected in the refined models had a more balanced presence, improving the reliability and generalizability of the models in subsequent phases.

Figure 2 presents ten activities with the highest weight. Those representing less than 3% of their weight within the dataset were grouped under the label “Other”. However, of the 10 activities initially selected, only 7 were considered because 3 of the 10 activities were only present in a single dataset. At the same time, the other 7 were found in several of the 23 datasets selected at the beginning of the study. Therefore, the activities finally considered are those presented in Table 4.

The following refined models were developed to guarantee a more rigorous analysis:

- Refined model with seven activities: This model considers only the seven activities selected in Table 4.
- Refined model with five activities: In this case, the climbing and descending stairs activities were excluded because although they were part of the dataset, they provided less information and had a lower classification performance than the other activities. The low performance created a problem in the overall performance of the model. These refined models focus on selecting a specific number of activities for evaluation to improve the comparative analysis between SL and BL approaches.

### 2.3. Model Evaluation Method

For the model evaluation phase, three scenarios were proposed:

- Scenario 1: BL classification. The first scenario consisted of classifying activities for each dataset’s rows and calculating the Accuracy, Precision, and F1 metrics based on the classifications made and the original activities.
- Scenario 2: SL classification. The scenario is the same as the previous one, with the difference that the SL models were used in this scenario.
- Scenario 3: incremental SL classification. In this scenario, the datasets were assumed to be already labeled as the model expected. Therefore, after each classification, the algorithm was fed the provided labels to reinforce its learning and, in theory, adapt to the provided samples.

#### 2.3.1. Model Evaluation Details

Four ADL datasets found in the literature (presented in Table 3) were used to perform the model evaluation. The four datasets were classified as external and internal datasets:

- External datasets: These are datasets with untrained models that recreate values from a “real” environment. In this categorization, the selected datasets were datasets with IDs 1 and 2. These datasets are not part of this study’s comprehensive, integrated dataset.
- Internal datasets: These are datasets with trained models. They were used to test the data in a more appropriate simulation. The selected datasets were datasets with IDs 3 and 4. These datasets are part of this study’s comprehensive, integrated dataset.

#### 2.3.2. Model Approval

The following considerations were taken into account for the approval of the algorithm to be used:

- Model objective.
- The model’s resource consumption.

### 2.4. Application Deployment Method

To successfully create the mobile application, named “Jaida”, the following tasks were required in the deployment, monitoring, and maintenance plan:

- Definition of the mobile application design principles.
- Definition of the mobile application environment.

#### 2.4.1. Principles of Mobile App Design Method

The first version of the “Jaida” mobile system was developed in Flutter [27] with a backend in Flask [28]. In addition, the mobile system was deployed on a micro instance of Amazon Web Services (AWS) EC2 technology [29]. Below are some of the criteria considered:

- Efficient and fast display of results.
- Intuitive navigation.
- UX-B1. The app complies with Android design guidelines and uses common user interface (UI) patterns and icons.
- PS-V2. The app displays text and blocks of text acceptably.

#### 2.4.2. Mobile Application Environment

Jaida is a mobile application whose APK allows it to run on any mobile device. It uses AWS to ensure minimum security policies on the server, and its interface follows the basic principles of mobile application design. The backend was developed in Flask 3.0 to be compatible with Python 3.11, and the application interaction was implemented with Flutter.

The system supports CSV files with specific columns that must maintain consistent names and positions. These files are processed using the app_caractersticas.py file to extract features and condense every 500 rows into one. Although segmentation of the test datasets is not necessary before uploading them to the system, it is recommended that it be performed.

Feature extraction organizes labeled accelerometer and gyroscope data, and a sample dataset is available in the GitHub 3.4 repository for reference. Users can upload datasets, select activities to classify, and obtain corresponding predictions, comparing the classified activity with the prediction made by the model in this first version of Jaida.

## 3. Results

### 3.1. Modelling Results

Once the models were trained for BL and SL, we evaluated their performance using metrics such as accuracy, AUC, and F1 score. These metrics are fundamental in supervised learning problems and allow us to analyze the ability of the models to classify instances within the datasets used correctly.

#### 3.1.1. BL Models

For this case, the cross-validation technique was applied to evaluate the model with subsets of the dataset [30], obtaining a more accurate performance estimation and reducing biases. The StratifiedKFold library was used to implement this technique, setting the seed to randomize the dataset. Once the randomization was completed, a five-fold (k = 5) cross-validation strategy was defined, splitting the dataset into five equal parts. This splitting leads to the model being trained and evaluated five times, alternating between one test fold and four training folds, rotating to cover all folds. The results obtained for each model are presented in Table 5.

Table 5 shows that the Naive Bayes and Support Vector Machine (SVM) algorithms were not included due to specific problems during their implementation. Naive Bayes was not implemented because the MultinomialNB algorithm does not support negative values in the input data, which are frequent in accelerometer and gyroscope measurements. On the other hand, SVM had an excessively long run time (approximately 15 h in Google Colab), which made it unfeasible for this study.

#### 3.1.2. SL Models

Three options were implemented to assess the model’s ability to adapt to new data and understand how they perform in different scenarios, giving proximity to real-world applications.

- First Option: Training and evaluation with the full dataset. The complete dataset was used, which was prepared as a continuous data stream using the “DataStream” class of the scikit-multiflow library. This preparation converts the dataset into a continuous sequence of samples, facilitating the model’s training using a classifier specific to each algorithm. A loop was implemented in the model training process that runs until reaching a maximum of 250,000 samples. In each iteration of this loop, the next sample was extracted from the data stream and partially used to update the model. Finally, the model’s performance was evaluated on the same dataset. This initial evaluation provides a measure of the initial performance of the model, thus allowing us to determine its ability to learn and generalize patterns in the training data.
- Second Option: Testing on a new dataset. After training the model with the final dataset, its adaptability was evaluated on a different dataset, which was not used during training. So, the previously trained model is loaded from a pkl file using the function “joblib.load()”. Then, a new dataset named “new_df“ is used to generate a new data stream using the DataStream class. This new data stream and the loaded model are passed to the previously configured evaluator. Finally, the evaluator method was used to process the data stream and the model, thus verifying its ability to adapt to new samples.
- Third Option: Training with 70% and Testing with 30%. Two datasets called dataset_training and dataset_testing were used. During the training phase, dataset_training was used to train the classifier, generating a data stream from this set, whose samples feed the learning model. The remaining 30% of the original dataset (dataset_testing) was used in the test phase. Once trained, the model was loaded, and its performance was evaluated. To carry this out, a data stream was created with dataset_testing, samples of which are used to make predictions using the trained model.

Finally, performance metrics such as accuracy, F1-score, and AUC were calculated, evaluating the training process with 70% of the data and the test with 30%. This approach seeks to simulate a realistic scenario in which the model is trained on a portion of the data and tested on completely new data. This allows for estimating the model’s performance in unknown situations and assessing its generalization and adaptability.

Once the three options were evaluated (First, Second, and Third Options), the low level of accuracy for the HoeffdingTreeClassifier algorithm was evident in the second case, as shown in Table 6, so it was decided to discard this case for analysis in the following three algorithms. Furthermore, due to the similarity of results in the metrics between Option 1 (complete dataset) and Option 3 (70% training and 30%), it was decided to test only Option 3 (70% training and 30%) for the following two missing models, as shown in Table 7.

The results obtained for both the BL and SL models fell short of the expected results. Of the five BL models that were fully compiled, only the Random Forest model achieved an accuracy of 72% and an F1-score of 68%. Although all models showed a high AUC (Area Under the Curve) value, indicating that the model can distinguish between classes, the accuracy was rather low for the most part. This result could be due to several factors, such as a class imbalance and problems with the feature set. On the other hand, out of the four SL models compiled, three have an accuracy higher than 70% in case number 3, which is the most relevant.

#### 3.1.3. Evaluation of PMWORV and PMWRV Models

The previous test plan, described in Section 2.2.3—Preliminary Models, involved the PMWORV models. Five models were compiled for BL, as detailed in Table 1, covering the 3 types of datasets mentioned, resulting in 15 models developed for BL. On the other hand, four models were compiled for SL, as shown in Table 2, for each of the 3 datasets, accounting for 12 models developed for SL. Together, this represents 27 models in the first test plan.

In this test, which was performed with the PMWRV algorithms, 2 models for BL and 2 for SL were used exclusively with the best-performing dataset, leading to a total of 31 models developed. We detail the results experienced in the evaluation phase, which are broken down as follows.

##### Evaluation of PMWORV

- Evaluation of PMWORV in BL: Table 8 presents the results obtained for the five BL models compiled on the three datasets analyzed. Table 8 presents most models, excluding “Model only dataset 2”, which shows a markedly superior performance. This result confirms the low reliability of dataset 2 data. It is collected in an uncontrolled environment and suffers from higher data uncertainty, given that the users themselves assign the activity labels. In addition, a significant difference in the performance of the models is highlighted. Specifically, the Random Forest model performed better than the other models evaluated.

- Evaluation of PMWORV in SL at 70%. Table 9 reports the results of the SL algorithms on 70% of the data. Good performance is evident in all models, except for Naive Bayes, both in terms of performance and size, highlighting the significant advantage of SL over BL. However, some notable disadvantages are also observed; firstly, the training time is higher in the best-performing models. On the other hand, a disadvantage of the KNN model was identified: it is constantly affected by window variation in its performance. As the window size increases, the amount of data stored in memory increases, negatively influencing its performance. After performing various tests, a window size of 100 was determined to provide the best accuracy.
- Evaluation of PMWORV in SL at 30%: Table 10 shows the results of the SL evaluation in the remaining 30% of the dataset for each model and dataset. The results revealed that the ARF and KNN algorithms exhibited metrics close to 100%. In contrast, the HT and NB algorithms experienced a notable decrease in performance, which is of concern given the ideal environment in which they were developed. Therefore, it is inferred that while the PMWORV models are useful for initial approximations to the SL and BL algorithms, they are unsuitable as algorithms to be incorporated into the applications “pkl” file.

##### Evaluation of Preliminary Models with Random Values (PMWRV)

Recognizing that the PMWRV models are not designed to operate in real conditions and are not optimal for the classification required by the application, the PMWORV was evaluated. During this phase, the activities were randomized to train the models inreal settings. It is important to note that the randomization does not affect the BL models, and the results remain consistent with those presented in Table 9. Therefore, the cross-validation technique was implemented in the PMWRV for BL to enrich the analysis.

- PMWRV models in BL with Cross Validation: Cross-validation aimed to determine how the model behaved in different dataset sections to avoid overfitting or bias. The results of this cross-validation are presented in Table 11. It is concluded that BL performs well in all activity options with random or non-random values.
- Evaluation of PMWRV in SL: PMWRV allows randomization in activities and values. In Table 12, we present the results of the SL models, which were trained and evaluated on the different datasets. It is observed that they are not optimal, suggesting that the randomization is not optimal. In addition, due to the limited memory in SL, the models fail to access a sufficient number of samples of the same activity to learn effectively, which prevents accurate classification of activities. Although the accuracy values in the three models are insufficient, the “model without dataset 2” has a considerably higher Area Under the Curve (AUC) and F-1 score compared to the full model and model-only dataset 2.

#### 3.1.4. Evaluation of Refined Models

The dataset that performed best in the models in the proposed options was the dataset_without_16. Therefore, the “Model without dataset 2” was chosen as the best model for both BL and SL. The most important fact for a refined model is to seek to improve generalization by reducing dimensionality. This is to reduce the risk of overfitting in the classification models due to the elimination of the classification models and the elimination of activities (such as those categorized as “OTHER” in Figure 2). The refined models were generated, one with five activities and the other with seven activities.

##### Evaluation of Refined BL Models

Table 13 presents the BL models with five and seven activities showing optimal performance in the metrics considered. The Random Forest (RF) model stood out as the best for BL, as it performed excellently except for the model with seven activities, where the RF model did not achieve a good percentage of RAM usage.

##### Evaluation of Refined SL Models

Table 14 presents the performance of the SL models. SL does not improve the performance compared to BL; however, SL does present an improvement in terms of the size of the model and the RAM usage.

The improvement in the performance of the algorithms due to the reduction in activities is remarkable compared to previous models, as the metrics were previously not good. Now, the results of both tables for both SL and BL exceed the 70th percentile threshold, even with randomized data. The hyperparameter of window size of the KNN algorithm had different tests with values of 100, 1000, 2000, 10,000, and all rows, where a directly proportional relationship was found between increasing the number of rows and increasing the algorithm’s performance. However, this also significantly increased the processing time. However, there was no great difference in the results between 10,000 and 20,000 rows, so to avoid increasing the training time, the decision was to use 10,000 rows.

#### 3.1.5. Analysis of Refined Models

##### Area Under the Curve (AUC) of the Refined Models

**Figure 3:**
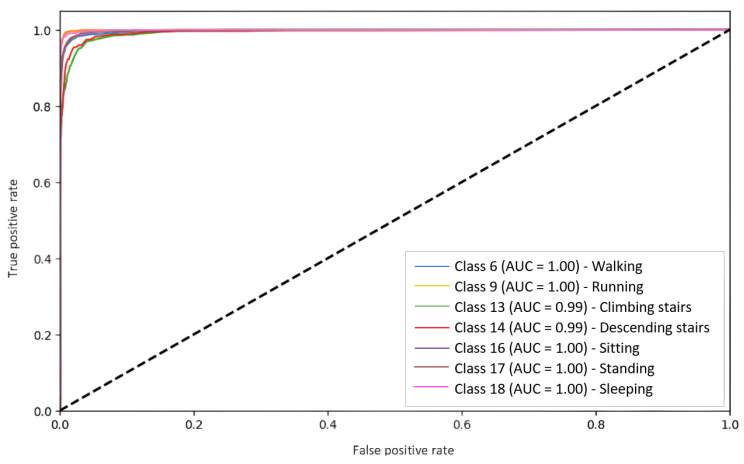
Receiver operating characteristic (ROC) plot of the BL RF algorithm for seven activities (as part of the BL refined models).

Figure 3 presents the performance of the BL RF model for seven activities (as part of the BL refined models). It clearly shows the performance of the models for each class corresponding to a specific activity, which reaches nearly 1.0.

**Figure 4:**
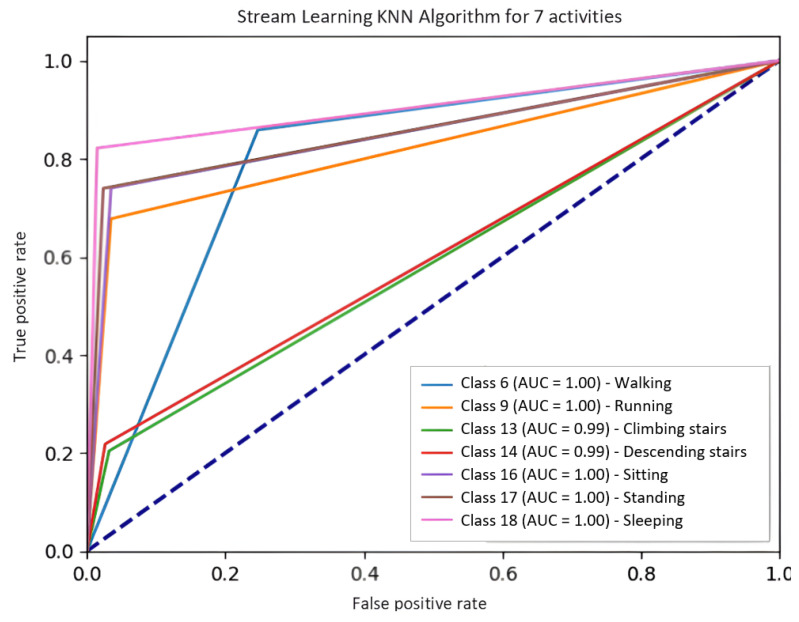
Receiver operating characteristic (ROC) plot of the KNN model for seven activities (as part of the SL refined models).

Figure 4 presents the performance of the KNN model for seven activities (as part of the SL refined models), with a significant decrease in the Area under the curve. However, the values are still quite acceptable, except for classes 13 and 14, which represent the activities of climbing and descending stairs. Finally, it is observed that the RF model (Figure 3) outperforms KNN (Figure 4) by a considerable difference in terms of AUC per class.

**Figure 5:**
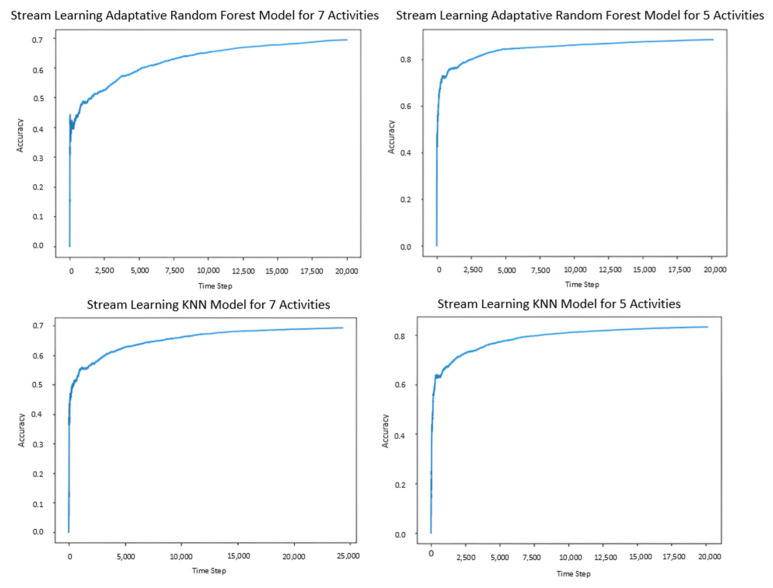
Learning curves of the refined SL models (RF and KNN with five and seven activities).

Figure 5 presents the learning curve of the SL refined models (ARF and KNN). It is possible to observe that, with only five activities, the accuracy improves significantly in both ARF and KNN models. Figure 5 also shows a faster improvement in accuracy, which supports that, at least in these SL algorithms, it is not advisable to classify many random activities. However, the values of the metrics with seven activities are still relatively high.

##### Modeling Analysis: First Differences Found Between BL and SL

- The SL models showed very poor performance when the data were not randomized. The SL models stored information in their memory from the last dataset activity with which they were trained. This meant that this was the only activity they could classify, so their performance was null once they tried to classify an activity other than this one.
- The BL models had no significant impact when trained with randomized datasets. This is because their distributed batch training allows the entire dataset to be stored.
- When the models were trained on an unstructured dataset with many classes, the SL models still presented difficulty predicting activities. This was because the more classes they were given, the worse their results were. BL, on the other hand, still performed well even if the number of classes was large.
- The BL models had a higher file weight and RAM usage than the SL models.
- The refined SL models performed better because there were fewer classes to store and no longer an imbalance of classes to cause problems in classification. This made both BL and SL compliant so that they could be evaluated with test datasets.
- Although the RF models offered a great improvement over models such as Decision Tree (DT) (for BL) or KNN (for SL), they generated significantly heavier models and consumed more RAM during training.
- In the ROC plots, the BL results were extremely close to 1.0 in each class, i.e., they achieved almost perfect predictions. However, the SL models performed fairly well but suffered when stair-climbing and descending activities were added

### 3.2. Model Evaluation

#### 3.2.1. Evaluation of Scenario 1: BL Classification

RF and DT algorithms were chosen to evaluate this scenario because of their better performance during the training phase. The most significant metrics that were taken into account were accuracy and precision. The accuracy metric was used to assess the classification and class imbalance.

- Evaluation of external datasets in BL model. The BL models showed considerably decreased accuracy and precision when tested with external datasets. In the analysis by activity, going up and down stairs had very low metric values. More precisely, with the dataset with ID 1 (in Table 3), the walking and standing activities were classified correctly. In dataset 2, the only activity that was correctly classified was sitting.
- Evaluation of internal datasets in BL model. Activities such as “walking” or “climbing stairs” with external datasets had a classification with a tendency to 0%; meanwhile, the known datasets have a percentage close to 90%. It is also highlighted that, although the two internal datasets present excellent results, the dataset with ID 3 (in Table 3) obtains a considerable improvement because it does have accelerometer and gyroscope information. In contrast, the dataset with ID 4 (in Table 3) only has accelerometer values.

In the accuracy metric, all BL internal models have high values.

#### 3.2.2. Evaluation of Scenario 2: SL Classification

The two best algorithms found were used to evaluate the SL classification model: ARF and KNN. The metrics considered were accuracy, precision, and F1, which allow for the correct evaluation of class imbalance classification. The external and internal models were analyzed.

- Evaluating external models in SL. The SL models classified dataset activities and/or information with which they had not been previously trained performed better than BL models. Results with the ARF algorithm outperformed those obtained in KNN. The best metric was precision, achieved in both datasets with ID 1 and 2 (in Table 3), classifying four and five activities, respectively, in ARF. However, it is important to mention that accuracy in dataset with ID 1, by ranking four activities, still does not perform well. It can be seen that climbing and descending stairs perform poorly.
- Evaluating internal models in SL. The SL models with which they were trained showed a decrease in performance compared to when the information to be classified is unknown. This decrease is due to the limited memory of SL, which cannot store all the information of the previously trained datasets.

#### 3.2.3. Evaluation of Scenario 3: Incremental Classification of SL

For scenario 3, incremental SL classification, only the best SL algorithm, ARF, was used since it proved to be the most efficient in accuracy and overall performance during the initial evaluation. When incremental SL learning was performed, the external and internal datasets presented a considerable improvement compared to the previous results. The main difference between classification and incremental classification in SL is that, in the first scenario, SL classifies activities based on a set of received features. In contrast, in the second scenario, SL categorizes activities and continuously learns. It gives the option to learn, i.e., it is constantly being fed back and trained.

At the activity level, as in the SL classification, the incremental classification performs better with external than internal data. This is due to SL’s limited memory. It is again rectified that dataset 2 has a high lack of reliability with its values; even in the SL incremental classification, it still presents low values compared to the rest.

**Figure 6:**
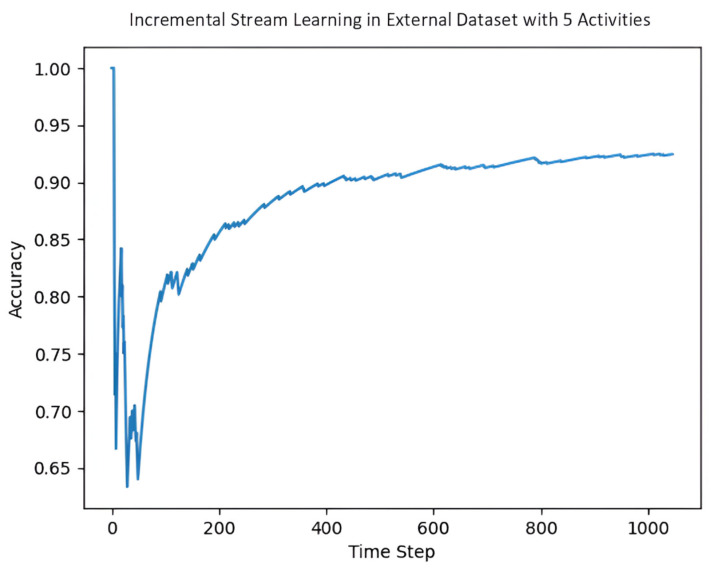
Incremental SL in an external dataset (Dataset with ID 1) with five activities.

BL models do not have the functionality to train incrementally, which is one of the unique advantages of SL models. Figure 6 shows the incremental SL learning on the external dataset with ID 1 with the RF algorithm.

#### 3.2.4. Model Selection

The considerations for choosing the model to be used in the deployment phase are presented below.

- The model is intended for classification in an environment with unknown data. To ensure that the model was trained correctly without a large number of classes, it is recommended that SL be chosen with its incremental function.
- If the model is lightweight, SL is a suitable choice. However, if many activities need to be trained and classified using the same or a similar trained model, BL may be more appropriate. The BL algorithm also needs to meet certain criteria:
  1. If there are fairly good computational resources and the goal is to improve accuracy, RF would be recommended in BL.
  2. If insufficient computational resources or a higher prediction speed is desired, DT would be recommended in BL.
- BL is an excellent option in business cases where the data will not change too much in the future. SL is recommended in business cases where it is foreseen that the amount of data will not always be the same as the data that were trained and that there will be a certain amount of new data every so often.

The SL classification model was selected to implement the application in the next stage of the methodology due to its accuracy and handling of RAM resources.

### 3.3. Application Deployment

#### 3.3.1. Principles of Mobile App Design

This mobile system followed Google’s mobile application design principles effectively and met the key criteria for successful navigation and exploration. The following is a display of the fulfillment of the above requirements.

- Results are efficiently and quickly displayed. Jaida presents its main features and new functions in appropriate contexts, guiding users to what they need with an obvious call to action, such as “Upload CSV” and “Generate Data”.
- Intuitive navigation. The application allows users to go back one step instead of returning to the beginning in case corrections, or changes are needed, avoiding data loss and reducing possible user frustrations by providing a smoother process.
- UX-B1. The app complies with Android design guidelines and uses common user interface (UI) patterns and icons.
- PS-V2. The app displays text and blocks of text acceptably.

Other important criteria that were fully met were no intrusive permissions, compatibility with standard system navigation via the “Back” button, including text labels for icons, and ease of use.

#### 3.3.2. Mobile Application Functionalities

The Jaida provides the following functionalities:

- Users have the facility to test the mobile system with a preloaded dataset and/or upload other datasets that meet the necessary characteristics to classify activities through its interface.
- Users can use a first test version and upload a CSV file (the requirements for this CSV are on the app). Then, it is possible to simulate these data to classify daily living activities
- Using a simple button, the user can randomly generate activity values with a dataset already preloaded by default.

In the first interface of the mobile system, users can easily identify the name of the Jaida mobile system and its two enabled options to load a CSV file and generate random data with which to make predictions on a default loaded dataset.

The second interface of the mobile system allows the user to identify whether the prediction matches the expected one easily.

## 4. Discussion

This study compared the performance of BL and SL algorithms in classifying ADL. The results highlighted that while BL achieved high accuracy in controlled environments, SL proved more efficient and adaptable in dynamic scenarios due to its incremental learning capability. Additionally, reducing the number of activities significantly improved performance in both approaches, underscoring the importance of appropriate data preprocessing.

SL’s superiority in dynamic scenarios reaffirms its suitability for real-time mobile applications. Conversely, BL’s robustness in controlled settings makes it a reliable option for predictable tasks. This dichotomy emphasizes that each method has specific applications depending on problem requirements. Furthermore, issues such as class imbalance highlight the need for more robust approaches to ensure accurate and reliable models.

The findings indicate significant potential for implementing SL in practical applications, particularly mobile devices. This would improve efficiency and facilitate autonomous activity detection and classification in real-world contexts. The results also underscore the importance of carefully validating and selecting datasets due to their direct impact on model accuracy.

Evaluating computational resource consumption in the analyzed models highlights a key consideration in real-world implementation, particularly for mobile and embedded systems. While BL models provide high accuracy, their elevated memory and processing demands can limit their deployment in resource-constrained environments. SL models, however, demonstrate a significant advantage due to their efficient memory management and adaptability to dynamic data streams, making them ideal for real-time applications. This distinction underscores the importance of selecting models based on accuracy and their ability to function within technological constraints, especially in devices where storage and energy consumption are critical factors.

This paper significantly advances personalized medicine by effectively addressing challenges within the pHealth ecosystem through implementing SL. This innovative approach enables real-time adaptation to patient behaviors, particularly in response to aging-related mobility changes. By integrating SL with Smartphone-based HAR (SHAR), we ensure that sensitive data are processed locally on the phone, enhancing patient autonomy.

A critical advantage of this method is its ability to precisely classify ADL, facilitating the early detection of health issues such as frailty or cognitive decline by identifying subtle deviations in routine behavior. The mobile app “Jaida” stands out as a prototype of how personalized health tools can provide real-time insights to users and caregivers, empowering self-monitoring and enabling the customization of care plans.

This work faced data quality limitations, particularly from a dominant dataset with inconsistencies, which may have introduced biases and reduced model generalization. Furthermore, complex activities were not deeply explored, which could provide new insights into SL and BL capabilities in more challenging applications.

For future research, it is recommended that the diversity and quality of datasets be increased, ensuring balanced classes and consistent data, with hybrid approaches combining SL with Deep Learning being explored to improve adaptability and pattern analysis, and studies on wearables being conducted to validate SL’s applicability in contexts with even stricter computational constraints.

## 5. Conclusions

The scientific community has shown a growing interest in patients’ daily behavior, which strongly impacts his/her personal health. This has motivated research on classifying ADLs using SL models on mobile devices.

The results obtained in this work allow us to identify the principal differences between models using BL algorithms and SL algorithms (with various options) in various contexts. This research’s contributions serve as a basis for future work on ADL with SL models and its application in other areas of healthcare.

The incremental SL function stands out for its ability to adapt to changes in various situations and projects. Our research has shown that this approach (incremental SL) significantly improves model performance, which is why it would be important in contexts of constant change. While BL models can efficiently manage data with diverse classes, their performance can be affected by unexpected changes or increased data flow. In contrast, SL models adapt better to new data, although their limited memory for storing classes can be challenging without clear objectives. Finally, using the CRISP-DM methodology has effectively guided this work, confirming its suitability for data science projects by structuring and grounding each research phase.

## Figures and Tables

**Table 1:** BL algorithms.

Algorithm	Description	Hyperparameters
Decision Tree	Splits data into branches and captures	criterion = ‘gini’, splitter = ‘best’,
	patterns. Can overfit.	max_depth = None, min_samples_split = 2,
		min_samples_leaf = 1,
		min_weight_fraction_leaf = 0.0,
		max_features = None, random_state = None)
Random Forest	Robust shaft assembly avoids	P 100, I 100, num-slots 1, K 0, M 1.0, V 0.001, S 1
	Over-adjustment.	
NaiveBayes	Simple, assumes independence between	A 0.5
	Variables. Good with categorical data.	
KNN	Classifies according to nearby neighbors.	N = 5, leaf-sizeint, default = 30
	Sensitive to noisy data.	
Logistic Regression	Models probability, linear. Limited for	R 10^−8^, M 1, num-decimal-places 4
	Non-linear data.	
GaussianNB	Naive Bayes with Gaussian distribution.	priors = None, var-smoothing = 10^−9^
	Effective with normal distributions.	
SVM	Separates classes with hyperplanes. Effective	Default parameters
	on linear and non-linear data.	

**Table 2:** SL algorithms.

Algorithm	Description	Hyperparameters
Hoeffding Tree	Adaptive tree with incremental changes. Low	max_byte_size = 33554432,
	memory and speed.	memory_period = 1000000, grace_period = 200,
		split_criterion = ‘info_gain’,
		split_confidence = 10^−7^, tie_threshold = 0.05,
		binary_split = False
Adaptive Random	Evolves with the data. High performance in	n_models = 10, max_features = “sqrt”,
Forest	continuous flow.	lambda = 6, metric = Accuracy: 0.00%,
		disable_weighted = False,
		drift_detector = ADWIN
Naive Bayes	Simple probability estimate. May not adapt to	Default parameters
	changes.	
KNN	Sorts with close neighbors. Sensitive to sudden changes.	N = 5, max_window_size = 1000, leaf_size = 30

**Table 3:** Individual datasets (of the 23 sources used in the configuration process of the final integrated dataset) were a relevant part of the evaluation process of the integrated dataset.

Dataset ID	Reference	Activities Detected
1	[23]	Walk, sit, stand, open the door, close the door, pour water, drink from a glass, brush your teeth, and wipe the table
2	[24]	Lying, sitting, standing in one place, standing, moving, walking, running, and cycling
3	[25]	Walking, jogging, stairs, sitting, standing, typing on the cell phone, brushing teeth, eating soup, eating packet potatoes, eating pasta, drinking from a drink, eating a sandwich, kicking a ball, playing catch with a tennis ball, dribbling (basketball), writing, clapping and folding clothes
4	[26]	Walking, Sitting, lying down, running, climbing stairs, going downstairs, standing, falling due to unconsciousness, falling due to a heart attack, and falling due to slipping while walking

**Table 4:** Activities in the dataset_without_2. A description of the activities and their corresponding labels.

Label	Activity
6	Walking
9	Running
13	Climbing stairs
14	Descending stairs
16	Sitting
17	Standing
18	Sleeping

**Table 5:** Metrics results for BL algorithms. A comparison of metrics obtained with baseline algorithms.

Model	Metric	Result	Cross Validation
	Accuracy	0.59	0.58
Decision Tree	AUC	0.76	–
	F1-score	0.59	–
	Accuracy	0.73	0.72
Random Forest	AUC	0.93	–
	F1-score	0.68	–
	Accuracy	0.19	0.1136
GaussianNB	AUC	0.73	–
	F1-score	0.06	–
	Accuracy	0.52	0.5368
KNN	AUC	0.81	–
	F1-score	0.51	–
	Accuracy	0.32	–
Logistic Regression	AUC	0.81	–
	F1-score	0.35	–

**Table 6:** Metrics for SL’s Hoeffding tree classifier and adaptive random forest classifier in the three options.

Hoeffding Tree Classifier						
First Option (Full Dataset)		Second Option (New Dataset)	Third Option (70% training and 30% evaluation)			
Accuracy	F1 Score	Accuracy	Accuracy (70%)	F1 Score (70%)	Accuracy (30%)	F1 Score (30%)
0.7801	0.6276	0.3924	0.7401	0.5950	0.4012	0.3722
**Adaptive Random Forest Classifier**						
First option (full dataset)		Second option (new dataset)	Third Option (70% training and 30% evaluation)			
Accuracy	F1 Score	Accuracy	Accuracy (70%)	F1 Score (70%)	Accuracy (30%)	F1 Score (30%)
0.9974	0.9613	-	0.9961	0.9481	0.9912	0.9241

**Table 7:** SL model metrics in third option (70–30%).

Naive Bayes			
Accuracy (70%)	F1 Score (70%)	Accuracy (30%)	F1 Score (30%)
0.6201	0.4476	0.2693	0.2059
**KNN**			
Accuracy (70%)	F1 Score (70%)	Accuracy (30%)	F1 Score (30%)
0.9947	0.9341	0.9840	0.8733

**Table 8:** PMWORV models in BL.

	BL						
PMWORV Models	Algorithms	Accuracy	AUC	F-1	Time (mm:ss)	Size	RAM
	Random Forest	0.7	0.93	0.68	04:00	3.26 GB	1.5 GB
Complete Model	Decision Tree	0.59	0.76	0.59	00:30	39.2 MB	0.07 GB
	Gaussian Naive Bayes	0.11	0.73	0.06	00:08	46 KB	2.10 GB
	KNN	0.53	0.81	0.51	03:20	63.8 MB	0.05 GB
	Logistic Regression	0.43	0.81	0.34	07:28	24 KB	0.1 GB
	Random Forest	0.92	1	0.92	00:41	2.96 GB	0.3 GB
Model without dataset 2	Decision Tree	0.82	0.9	0.82	00:13	2.9 MB	0.01 GB
	Gaussian Naive Bayes	0.35	0.9	0.31	00:07	35 KB	0 GB
	KNN	0.51	0.85	0.5	00:19	13.4 MB	0 GB
	Logistic Regression	0.45	0.9	0.4	01:34	19 KB	0 GB
	Random Forest	0.64	0.9	0.61	02:07	1.68 GB	1.5 GB
Model only dataset 2	Decision Tree	0.52	0.71	0.52	00:14	20.8 MB	0.2 GB
	Gaussian Naive Bayes	0.08	0.68	0.03	00:08	26 KB	1.02 GB
	KNN	0.55	0.79	0.53	02:07	50.4 MB	0.04 GB
	Logistic Regression	0.48	0.77	0.38	04:20	14 KB	0.04 GB

**Table 9:** PMWORV models in SL at 70%.

SL (70%)							
PMWORV Models	Algorithms	Accuracy	AUC	F-1	Time (mm:ss)	Size	RAM
Complete Model	Adaptative Random Forest	0.99	0.98	0.94	16:24	221 KB	0
	Hoeffding Tree	0.74	0.81	0.59	05:31	1.8 MB	0
	Naive Bayes	0.62	0.74	0.44	05:20	385 KB	0
	KNN	0.99	0.98	0.93	01:30	41 KB	0
Model without dataset 2	Adaptative Random Forest	0.98	0.98	0.93	05:21	331 KB	0
	Hoeffding Tree	0.8	0.87	0.71	02:33	730 KB	0
	Naive Bayes	0.65	0.81	0.60	01:20	291 KB	0
	KNN	0.98	0.97	0.92	00:45	41 KB	0
Model only dataset 2	Adaptative Random Forest	0.99	0.96	0.89	12:10	112 KB	0
	Hoeffding Tree	0.75	0.72	0.40	03:20	1.5 MB	0
	Naive Bayes	0.18	0.54	0.08	02:25	204 KB	0
	KNN	0.99	0.94	0.85	01:12	40 KB	0

**Table 10:** PMWORV models in SL at 30%.

SL (30%)							
PMWORV Models	Algorithms	Accuracy	AUC	F-1	Time (mm:ss)	Size	RAM
Complete Model	Adaptative Random Forest	0.99	0.975	0.92	07:36	Not applicable	0
	Hoeffding Tree	0.4	0.74	0.37	07:17		0
	Naive Bayes	0.269	0.65	0.2	06:27		0
	KNN	0.98	0.94	0.97	03:21		0
Model without dataset 2	Adaptative Random Forest	0.96	0.98	0.91	01:53		0
	Hoeffding Tree	0.55	0.81	0.53	03:01		0
	Naive Bayes	0.35	0.75	0.38	01:40		0
	KNN	0.94	0.92	0.83	01:10		0
Model only dataset 2	Adaptative Random Forest	0.99	0.93	0.83	05:19		0
	Hoeffding Tree	0.3	0.59	0.13	05:25		0
	Naive Bayes	0.291	0.55	0.05	02:45		0
	KNN	0.98	0.9	0.79	02:13		0

**Table 11:** PMWRV models with cross-validation.

BL Cross Validation								
PMWRV Models	Algorithms	Accuracy	Precision	AUC	F-1	Time (mm:ss)	Size	RAM
Complete Model	Random Forest	0.7	0.72	0.95	0.68	15:09	Not applicable	2.5 GB
	Decision Tree	0.58	0.58	0.78	0.58	02:09		0.35 GB
Model without dataset 2	Random Forest	0.91	0.91	1.0	0.91	03:55		1.01 GB
	Decision Tree	0.81	0.81	0.88	0.81	00:30		0.17 GB
Model only dataset 2	Random Forest	0.64	0.66	0.85	0.60	08:16		2.2 GB
	Decision Tree	0.52	0.52	0.67	0.52	01:17		0.16 GB

**Table 12:** PMWRV models in SL.

SL								
PMWRV Models	Algorithms	Accuracy	Precision	AUC	F-1	Time (mm:ss)	Size	RAM
Complete Model	Random Forest	0.38	0.35	0.58	0.20	85:12	2 MB	0 GB
	KNN	0.43	0.21	0.57	0.16	49:54	3.8 MB	0 GB
Model without dataset 2	Random Forest	0.40	0.40	0.68	0.37	30:23	1.1 MB	0 GB
	KNN	0.41	0.30	0.62	0.26	41:52	3.8 MB	0 GB
Model only dataset 2	Random Forest	0.47	0.25	0.53	0.08	48:40	2.6 MB	0 GB
	KNN	0.48	0.21	0.56	0.15	50:06	3.8 MB	0 GB

**Table 13:** Evaluation of refined BL models.

BL								
Refined Models	Algorithms	Accuracy	Precision	AUC	F-1	Time (mm:ss)	Size	RAM
5 Activities	Random Forest	0.98	0.98	1.00	0.98	01:10	9 MB	0.12 GB
	Decision Tree	0.95	0.95	1.00	0.95	00:10	89 KB	0.02 GB
7 Activities	Random Forest	0.95	0.95	0.97	0.95	01:40	26 MB	0.4 GB
	Decision Tree	0.89	0.89	0.93	0.89	00:15	257 KB	0.05 GB

**Table 14:** Evaluation of refined SL models.

SL								
Refined Models	Algorithms	Accuracy	Precision	AUC	F-1	Time (mm:ss)	Size	RAM
5 Activities	Random Forest	0.88	0.72	0.90	0.71	00:30	1.6 MB	0
	KNN	0.83	0.68	0.87	0.67	19:10	3.8 MB	0
7 Activities	Random Forest	0.69	0.6179	0.76	0.517	00:42	914 KB	0
	KNN	0.70	0.59	0.79	0.56	12:34	9.3 MB	0

## Data Availability

The data presented in this study are available on request from the corresponding author.
